# Event and Comment

**Published:** 1921-01

**Authors:** 


### DENTAL REGISTER

THE MAGAZINE OF DENTISTRY

Vol. LXXV JANUARY, 1921 No. 1

### EVENT AND COMMENT

**Preserving the Pulps.** Dr. C. N. Johnson, in *Oral Health,* makes this comment on the work which Dr. T. W. Maves, of Minneapolis, is doing in the preparation of vital teeth for bridgework. He says: "Dr. Maves believes in pulp conservation, and has worked out methods which require less cutting away of tooth structure for abutment preparation than other teachers of crown and bridge technic, and thereby causes only a minimum amount of pulp disturbance in this work."

"It must be noted by every observant practitioner that there is a growing tendency on the part of the profession to avoid pulp destruction wherever possible. The altogether routine and off-hand manner in which pulps have been destroyed in the past will no longer pass muster for good dentistry, and while the methods of pulp canal filling have been greatly improved, yet there is no filling for the canal of a tooth that can in any way equal a good, healthy pulp itself."

This brings up the old question of how far we should encourage those operations which threaten live pulps in healthy teeth. How many chances can we take with teeth whose pulps have been subjected to irritations because of encroaching caries and other causes which make such teeth of lower vital resistance? The pulps of teeth are wonderfully cared for by nature, but they have been and are still subjected to such heroic and unnatural treatment that one wonders how they can stand for these injuries without more serious consequences. We have been accustomed to think that so long as the calcified protection does not too seriously encroach upon the sensitive tissues, the tooth itself as an organ is safe. This seems not only logical but is in harmony with much of our past practice.

Isn't it possible that much of the criticism of patients because of sensitive and irritable teeth generally arises from the discomforts of patients which could have been avoided by more careful preservation of the physiological functions of the teeth? The ignorant and reckless carelessness which characterizes much of our technical treatment is astonishing when we stop to consider how beautiful and important the tooth really is.

If we could ever get a realizing sense of the value of the human teeth, from every standpoint, it would seem that we would have a higher sense of their importance and we should then do our part more willingly in their conservation. It would restrain us at least from ruthless mutilation and unrestrained abandonment by the operators and extractors.

We are now struggling to find ways and means for making the best repair efforts practicable, and we are doing this because we know no better way to relieve humane discomfort and much actual misery. We are doing a good work from many viewpoints, and we believe we are making some desirable progress. When we stop to view the progress made, we can not fail to notice that more is needed to be done than it is probable the best the dental profession can ever accomplish, considering the constant increasing needs which we see little hope we shall ever overcome. The task seems hopeless unless some new processes are devised to help us out. The point we want to call attention to is that we must do something we have not tried, that might open a new and better way. Are we thinking or working on any better ways that may make our problems more successful or even more promising? At present we are studying the pathologic and technical wholly. Is there any physiology that promises more of health and dental happiness? Is there much in prophylaxis that seems likely to materialize into real preventive dentistry?

